# Habitat area and climate stability determine geographical variation in plant species range sizes

**DOI:** 10.1111/ele.12184

**Published:** 2013-10-03

**Authors:** Naia Morueta-Holme, Brian J Enquist, Brian J McGill, Brad Boyle, Peter M Jørgensen, Jeffrey E Ott, Robert K Peet, Irena Šímová, Lindsey L Sloat, Barbara Thiers, Cyrille Violle, Susan K Wiser, Steven Dolins, John C Donoghue, Nathan J B Kraft, Jim Regetz, Mark Schildhauer, Nick Spencer, Jens-Christian Svenning

**Affiliations:** 1 Ecoinformatics and Biodiversity Group, Department of Bioscience, Aarhus UniversityDK-8000, Aarhus C, Denmark; 2 Department of Ecology and Evolutionary Biology, University of ArizonaTucson, AZ, 85721, USA; 3 The Santa Fe Institute, USA1399 Hyde Park Rd, Santa Fe, NM, 87501, USA; 4 School of Biology and Ecology/Sustainability Solutions Initiative, University of MaineOrono, ME, 04469, USA; 5 The iPlant CollaborativeThomas W. Keating Bioresearch Building, 1657 East Helen Street, Tucson, AZ, 85721, USA; 6 Missouri Botanical GardenP.O. Box 299, St. Louis, MO, 63166-0299, USA; 7 Department of Biology, University of North CarolinaChapel Hill, NC, 27599-3280, USA; 8 Center for Theoretical Study, Charles University in Prague and Academy of Sciences of the Czech RepublicJilská 1, 110 00, Praha, Czech Republic; 9 New York Botanical Garden2900 Southern Blvd, Bronx, NY, 10458-5126, USA; 10 CNRS, UMR5175, Centre d'Ecologie Fonctionnelle et EvolutiveF-34000, Montpellier, France; 11 Landcare ResearchP.O. Box 69040, Lincoln 7640, New Zealand; 12 Department of Computer Science and Information Systems, Bradley University1501 W Bradley Avenue, Peoria, IL, 61625, USA; 13 Department of Biology, University of MarylandCollege Park, MD, 20742, USA; 14 National Center for Ecological Analysis and Synthesis, University of California735 State Street, Suite 300, Santa Barbara, CA 93101, USA

**Keywords:** Climate stability, geographical range size, habitat area, New World, plants, Rapoport's rule

## Abstract

Despite being a fundamental aspect of biodiversity, little is known about what controls species range sizes. This is especially the case for hyperdiverse organisms such as plants. We use the largest botanical data set assembled to date to quantify geographical variation in range size for ∼ 85 000 plant species across the New World. We assess prominent hypothesised range-size controls, finding that plant range sizes are codetermined by habitat area and long- and short-term climate stability. Strong short- and long-term climate instability in large parts of North America, including past glaciations, are associated with broad-ranged species. In contrast, small habitat areas and a stable climate characterise areas with high concentrations of small-ranged species in the Andes, Central America and the Brazilian Atlantic Rainforest region. The joint roles of area and climate stability strengthen concerns over the potential effects of future climate change and habitat loss on biodiversity.

## Introduction

A species’ geographical range is a basic unit of comparative biology, biogeography and macroecology (Brown *et al*. 1996; Gaston 2003). Range size varies across species by several orders of magnitude (Willis 1922; Brown *et al*. 1996), and the spatial distribution of small- and broad-ranged species is uneven (Pagel *et al*. 1991; Jetz *et al*. 2004; Graves & Rahbek 2005; Morin & Lechowicz 2011; Sandel *et al*. 2011). Proposed linkages between the distribution of range sizes and species richness (Stevens 1989; Graves & Rahbek 2005) suggest that understanding the drivers of geographical variation in range sizes may be key to revealing what shapes species diversity. Understanding range-size distributions and determinants is, furthermore, essential for identifying regions of high conservation importance (Myers *et al*. 2000) and their sensitivity to anthropogenic environmental change (Ohlemüller *et al*. 2008). Further, range size is negatively related to extinction risk (Gaston 2003). However, nearly all of the above studies of range size are for vertebrate groups with a maximum diversity of a few thousand species. As a result, we have minimal knowledge of range-size variation and determinants in hyper-diverse groups like plants and insects.

Variation in species’ range size may reflect a variety of contrasting ecological, evolutionary and historical factors via speciation, extinction and range transformations (Gaston 1998). Two major mechanisms can be hypothesised: climatic stability and habitat area. Large range sizes have been associated with increased long- or short-term climatic instability whereas small ranges are concentrated in areas with stable climate (Janzen 1967; Stevens 1989; Jansson 2003; Sandel *et al*. 2011). Climate instability is usually proposed to select for large range sizes via intraannual variability, as invoked in Rapoport's rule (Stevens 1989). However, long-term temporal instability may also select for large range sizes, notably via orbitally induced climatic variability on 10^4^–10^6^ year time scales (Dynesius & Jansson 2000). Indeed, long-term climatically unstable areas have been found to harbour lower proportions of small-range species (Jansson 2003; Sandel *et al*. 2011). This relation has been attributed to increased extinction of small-range species under climate change due to narrow climate tolerance, poor dispersal capability, small extent and/or smaller population size, as well as reduced speciation due to extinctions of incipiently speciating populations and gene-pool mixing (Dynesius & Jansson 2000; Sandel *et al*. 2011). Despite the larger timescale, the mechanism is thus similar to how intraannual variability affects individuals and populations.

Small range sizes have alternatively been associated with small habitat area such as small land area (Hawkins & Diniz-Filho 2006), rare environments (Ohlemüller *et al*. 2008) or small habitat fragments due to dispersal barriers (Hawkins & Diniz-Filho 2006). Land area has previously been proposed to explain differences in range size between continents (Letcher & Harvey 1994) and to cause small range sizes among mammals in southern South America (Ruggiero *et al*. 1998). In principle, locations surrounded by large land areas should harbour relatively more broad-ranged species due to a larger potential for expansion (Hawkins & Diniz-Filho 2006). Considering the role of climate as a range determinant (Brown 1995), an alternative way of representing habitat area is climate rarity. Areas with unusual climates compared to their surroundings are expected to host more small-ranged species, reflecting their restriction to these rare conditions (Brown & Gibson 1983; Ohlemüller *et al*. 2008). Finally, elevation range reflects the strength of climatic gradients and associated habitat changes within an area (Ruggiero & Hawkins 2008). Steep elevation-induced environmental gradients may limit the habitat available for a species and act as dispersal barriers between similar environments, effectively restricting range size. They may also buffer climate change by reducing the distance species must move to track climate change over time (i.e., by reducing the spatiotemporal climate-change velocity, Loarie *et al*. 2009), thus acting as a mechanism more linked to climatic stability rather than availability of habitat area. In either case, mountains will be associated with high proportions of small-ranged species (Hawkins & Diniz-Filho 2006).

The generality and relative importance of climate stability and habitat area in shaping the distribution of range sizes remain unresolved (Gaston *et al*. 1998; Weiser *et al*. 2007; Ohlemüller *et al*. 2008), especially in important and hyper-diverse organism groups such as vascular plants. Here, we use the largest botanical data set assembled to date to map and analyse range-size distributions of nearly 85 000 species of non-marine vascular plants (hereafter ‘plants’) across the New World (Enquist *et al*. 2009). Specifically, we examine geographical variation in range-size frequency distributions in assemblages of co-occurring species in 10 000 km^2^ grid cells, with range sizes estimated from species’ occurrence points. Similar studies are few and limited to animal groups or smaller areas (Graves & Rahbek 2005; Hawkins & Diniz-Filho 2006; Morin & Lechowicz 2011). We test the relative importance of the climate stability and habitat area hypotheses in driving general patterns of plant range-size distributions, and examine how these processes vary in importance between regions of the New World. Finally, we assess the conservation implications of our findings in light of ongoing anthropogenic environmental changes. Overall, our work provides the first comprehensive assessment of range-size patterns for a hemisphere-scale flora and the first test comparing the relative effects of short- and long-term climate stability and habitat area for the New World plants.

## Methods

### Species data

**Figure 1:**
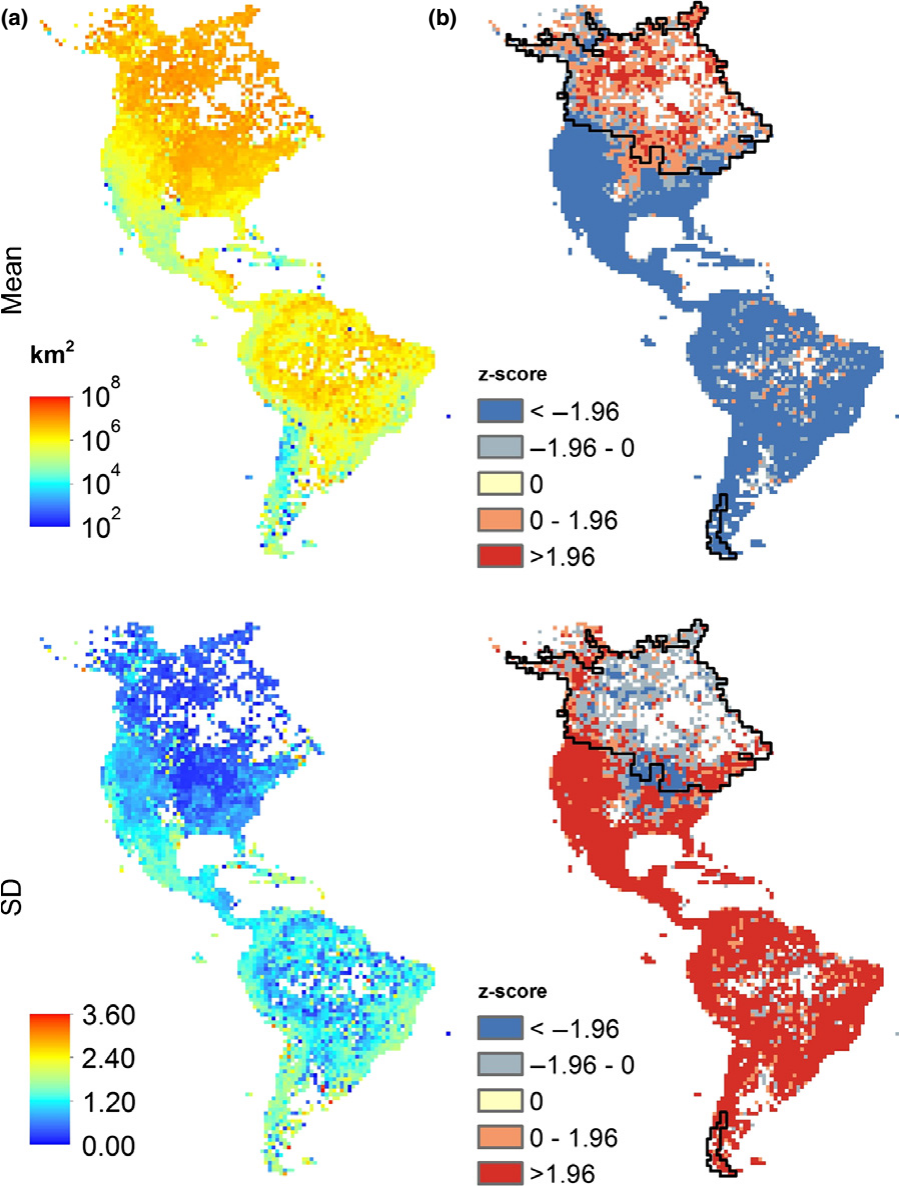
Maps for (a) range-size mean and variability (SD) of New World plants; and (b) deviations from random expectation. Cells with a value greater or lower than expected given observed species richness are coloured red or blue, respectively. Black line delimits glaciated areas during the Last Glacial Maximum.

We used the largest botanical database yet assembled for the New World (BIEN, the Botanical Information and Ecology Network) with 4 406 875 occurrence records for 84 899 plant species (Enquist *et al*. 2009; http://bien.nceas.ucsb.edu/bien/). All observations were assigned standardised taxon names using the Taxonomic Name Resolution Service (Boyle *et al*. 2013) and geographical locations were validated using the Global Administrative Areas data set version 2.0 (http://www.gadm.org, accessed on 11 May 2011). Cultivated occurrence records were excluded using original cultivated flags and locality descriptions. We projected all records (excluding Greenland) to a Lambert Azimuthal Equal Area co-ordinate system and calculated the range size of each species as the area in km^2^ of the convex hull encompassing all their New World records or, for species with < 3 records, as the summed area of all occupied grid cells with a grain size of 10 000 km^2^. Marine areas and large lakes were excluded from the convex hulls, which all had a minimum size of 10 000 km^2^. For each 10 000 km^2^ grid cell, we built a list of range sizes corresponding to species recorded within the cell. Hereafter, we call this per-cell distribution of range sizes the ‘range-size frequency distribution’. All analyses were done on log_10_-transformed range sizes due to the lognormal nature of both the per-pixel and global distributions of range sizes. We mapped range-size mean and SD (standard deviation) patterns based on the range-size frequency distribution for all unique species recorded in each cell, excluding cells with no records (Fig. 1). Thus, a cell with four species of range sizes 50 000, 10 000, 200 000 and 300 000 km^2^, respectively, would have mean (log_10_-transformed) range size 4.87 and SD 0.67. The range-size frequency distribution will be shaped by mechanisms such as evolution and dispersal that act directly on range size as well as mechanisms such as biotic interactions and environmental filters that influence which species are present in any given assemblage. This spatial assemblage approach has the advantage of better retaining geographical information than in methods comparing ranges among species (e.g. Pagel *et al*. 1991). While the mean quantifies the central tendency in an area's range sizes, the SD represents the variability of range sizes of species co-occurring in the same place.

Under the null hypothesis that the range-size frequency distribution should be equal for all cells independent of species richness or sampling effort, we mapped deviations from chance expectation for the mean and SD of range sizes, using the following randomisation approach: First, and because there is a higher chance of finding a broad-ranged species at any given cell, we weighted the probability of drawing a particular range size by its own area. For every cell, we then drew a sample equal to the observed number of species 1000 times from the overall range-size frequency distribution and compared this expected distribution to the observed in the cell. Mapping these *z*-scores, we were able to find areas that deviated significantly from a null randomisation (Fig. 1b).

### Explanatory variables

To test hypotheses on the drivers of spatial variation in range-size patterns, we included predictors representing climate stability in time and environmental variation in space (hereafter habitat area). We extracted data on current climate from the 5 arc-minute resolution WorldClim data set (Hijmans *et al*. 2005), projected and aggregated to a 10 000 km^2^ resolution. As a measure of present intraannual climatic stability, we used temperature and precipitation seasonality (TSEA and PSEA respectively). Following Sandel *et al*. (2011), longer time climate stability was represented by Late Quaternary climate-change velocity measured as the mean annual temperature velocity since the Last Glacial Maximum (21 000 year ago), corresponding to one of the strongest climatic shifts of the Quaternary. Climate-change velocity is thus a measure of the local temporal rate of geographical displacement of climatic conditions, calculated by dividing the temperature change over time by the local temperature change across space, which is lower where elevation gradients are present. The measure was based on estimates of past mean annual temperature from the Paleoclimate Modelling Intercomparison Project Phase II (Braconnot *et al*. 2007), using the mean of the CCSM3 (Collins *et al*. 2006) and MIROC3.2 (K-1 model developers 2004) simulations.

We represented habitat area by land area, climatic rarity and elevation range. We computed the land area measure of each cell by calculating the area of landmass available around it (excluding large lakes) within a 1800 km radius. This radius corresponded to the maximum inscribed circle of the land polygons. Thus, the land area measure had peaks close to the centre of North and South America respectively. Changing the radius for computing land area did not alter the modelling results (Table S3). Small-ranged species are expected to be concentrated in areas of rare climates (Ohlemüller *et al*. 2008). We developed a new measure of climate rarity taking into account all 19 climate layers from the WorldClim data set (Hijmans *et al*. 2005). To circumvent collinearity issues, we first ran a principal component analysis (PCA) on the 19 layers, transformed where appropriate to comply with the normality assumption, and standardised. The first two PCA axes captured 80.7 % of the climatic variation in the study area. Using these axes, we calculated the average Euclidian distance in climatic space between each cell and all other cells within a 1000 km radius. High values of the final measure correspond to cells that have rare climatic conditions compared to their neighbouring cells. This new climate rarity measure improves the measure used in Sandel *et al*. (2008) by being based on a broad range of climatic variables instead of only two. Elevation range was calculated by projecting 30′-resolution elevation data from the WorldClim data set (Hijmans *et al*. 2005) to equal area 1 km^2^ resolution and computing the difference between the minimum and maximum elevation found within each 10 000 km^2^ cell.

We considered latitude and data on productivity (annual mean NDVI across 1982-2000, downloaded from http://edit.csic.es/Soil-Vegetation-LandCover.html), but both were correlated with TSEA (Pearson's *r* = 0.813 and −0.703 respectively) and thus excluded from the analyses. Although productivity has been proposed to drive range-size distribution patterns in animal groups (e.g. Jetz & Rahbek 2002), it is unclear how this mechanism would work in plants. Further, it is still debated how it should be measured (Huston & Wolverton 2009), and choosing TSEA avoided the potential circularity issue of using a plant-based measure to predict plant range-size distributions.

Finally, we explored sensitivity to anthropogenic climate change by matching range-size spectrum types to predicted climate-change velocities for the 2080s under the A1B emissions scenario (Hijmans *et al*. 2005; Sandel *et al*. 2011).

### Data analysis

**Figure 2:**
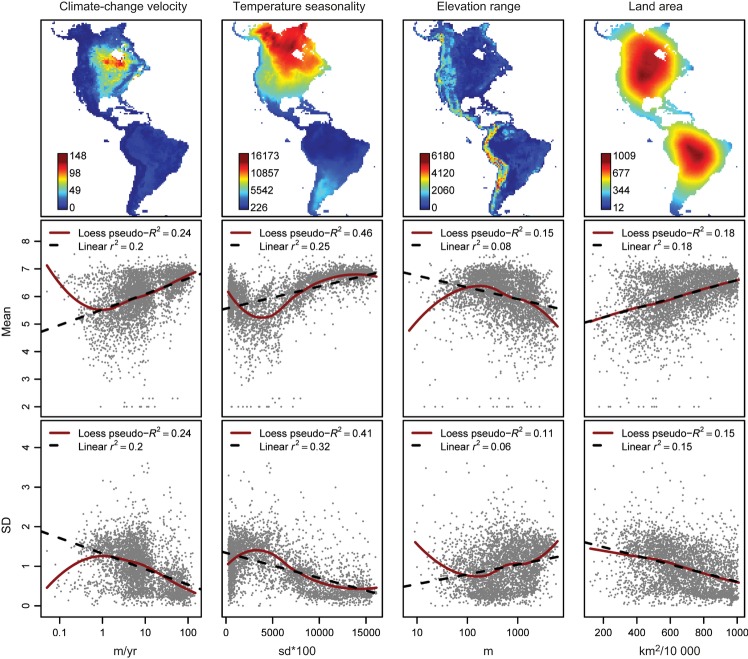
Maps of main potential predictors and their bivariate relationship to range-size mean and variability (SD). Linear and Gaussian local (LOESS, fitted with span = 0.75 and a quadratic term) regressions were fitted for all cells with at least one recorded species.

We tested the different hypotheses on the drivers of the range-size distributions using several approaches. First, we showed trends in the data using univariate non-spatial ordinary least squares (OLS) linear regressions and locally weighted regression (LOESS) to assess the relationship between the range-size mean and SD distribution patterns and each of the six single predictors (transformed where appropriate and standardised) (Fig. 2, Fig. S1). Second, we ran multiple OLS linear regressions using all predictor variables. The substantial amount of spatial autocorrelation that was left in the residuals of the OLS (Table S1) could potentially affect the parameter estimates and significance of statistical tests. We addressed this issue by incorporating all six predictors into simultaneous autoregressive (SAR) models, assuming the autoregressive process in the error term. It is not easy to choose the best specifications for SAR models *a priori*, because the amount of spatial autocorrelation varies among data sets (Kissling & Carl 2008). Therefore, we experimented with a range of distances (100, 200, 300, 400, 500, 1000 and 3000 km) and coding schemes for the spatial weights matrix (binary and row-standardised) to define the neighbourhood of each grid cell, and used the Akaike Information Criterion (AIC) and the minimum residual spatial autocorrelation (minRSA) to select the most appropriate SAR model for each response variable (Burnham & Anderson 2002). The final models selected had all row-standardised coding and neighbourhood distances of 300 km for models of mean range-size and 500 km for SD. The SAR models significantly decreased the amount of residual spatial autocorrelation with respect to the OLS models, and all had Moran's I < 0.1 in the first 20 distance classes (Table 1, Table S1). Model fit (*R*^2^) of the full models was assessed using squared Pearson correlation of predicted and observed values.

**Table 1:** Summary results for full SAR models explaining the mean and SD patterns for log_10_-transformed range sizes and variation partitioning (excluding the spatial component) of the two broad mechanisms, climate stability and habitat area

	Distance	AIC	minRSA	Max I	*R*^2^	V_total_	V_C_	V_H_	V_CH_
Mean	300	4989	0.232	0.053	0.690	0.364	0.134	0.057	0.173
SD	500	3435	0.174	0.058	0.520	0.383	0.186	0.028	0.169

Distance: radius (in km) used to define the neighbourhood matrix. AIC, Akaike's information criterion; minRSA, residual spatial autocorrelation (summed absolute Moran's I values of the first 20 distance classes); Max I, maximum Moran's I in the first 20 distance classes; *R*^2^, pseudo-*R*^2^, squared Pearson correlation between predicted and observed values; V_total_, variation (*R*^2^) explained in full models; Vc, unique contribution of climate stability; V_H_, unique contribution of habitat area; V_CH_, shared effect of climate stability and habitat area.

We evaluated all possible subsets of the full SAR model and used AIC values to quantify the support for each model. The Akaike weight (w) of each model can be interpreted as the probability that a specific model is the best in the candidate set for a response variable (Burnham & Anderson 2002). These weights allowed us to compute averaged parameter estimates across all models and estimate the relative importance of each predictor in explaining the range-size mean and SD patterns. The relative importance of the two broad mechanisms, climate stability and habitat area, was estimated using variation partitioning (Legendre & Legendre 1998). We used partial SAR and OLS models for climate stability (including climate-change velocity, PSEA and TSEA) and for habitat area (including elevation range, climate rarity and land area), and assessed the unique and shared contribution of each of the two broad mechanisms subtracting partial pseudo *R*^2^-values from the full model (Table 1, Table S1).

**Figure 3:**
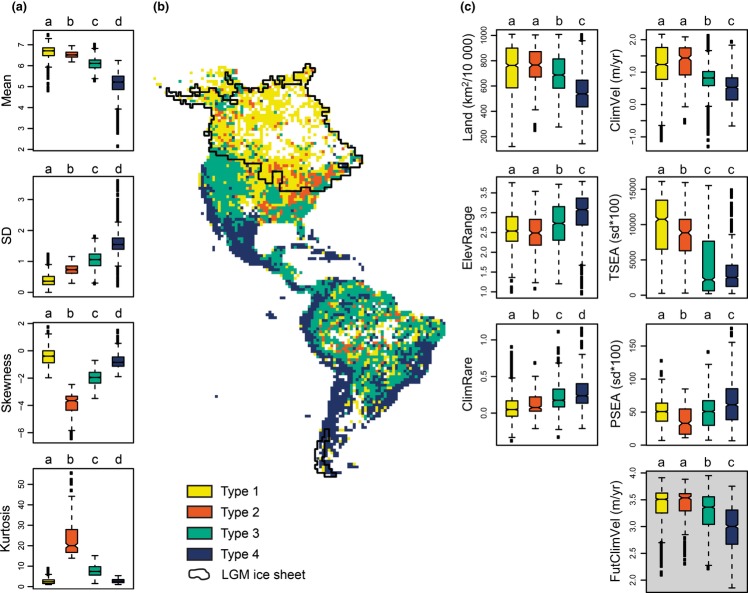
The four range-size spectrum types resulting from classifying each cell according to the shape of its range-size frequency distribution in a k-means cluster analysis. (a) Range-size characteristics of each spectrum type, (b) spatial distribution of spectrum types, (c) differences between types in predictor values and expected future climate-change velocity (grey box). Identical lower case letters above a given boxplot indicate groups not significantly different from each other (Mann–Whitney *U*-test, *P* < 0.001 with Bonferroni correction). Abbreviations as in Table 2.

We performed a k-means cluster analysis to classify all grid cells into spectrum types according to the shape of their assemblage range-size frequency distribution, considering the first four moments of the range-size spectrum, i.e. not just mean and SD, but also skewness and kurtosis (Fig. S2, S3). The skewness describes the asymmetry of the range-size frequency distribution, while the kurtosis describes its peakedness. Thus, the spectrum summarises the shape of the range-size frequency distribution. The classification allowed us to identify and map specific range-size spectrum types. We made boxplots and used Mann–Whitney *U*-tests with Bonferroni correction to test for differences among these spectrum types with respect to their range-size characteristics, the six predictor variables, and future climate-change velocity respectively (Fig. 3a, c).

All analyses were computed in R 2.15.3 (R Development Core Team 2013) and ArcGIS 10 (ESRI, Redlands, CA, USA).

## Results

The mean geographical range size for non-marine vascular plant species varied by seven orders of magnitude across grid cells in the New World. In North America, assemblage mean range size increased northward, whereas in South America it decreased southward away from the equator (Fig. 1a). The *R*^2^ values of the full SAR models were 0.690 for mean range size and 0.520 for range-size SD (Table 1). For mean range size, summed Akaike weights across SAR models were highest for temperature seasonality, climate-change velocity and land area, all with positive averaged standardised regression coefficients (Table 2). Temperature seasonality and land area had also the highest summed Akaike weights in models for range-size SD, but with negative coefficients. A third variable, elevation range, with a positive averaged regression coefficient, also had high importance for SD patterns (Table 2). Both for range-size mean and SD, regression coefficients of all supported variables in the SAR models retained their direction from the univariate models (Fig. 2; indicating there were no serious collinearity problems). Excluding the 21 978 rarest species with 10 000 km^2^ range sizes from the analysis provided the same results, indicating that they were not biased by the many species with few records (Table S4). Variation partitioning showed that climate stability and its combined effect with habitat area could account for most of the variation in range-size mean and SD (Table 1).

**Table 2:** Averaged standardised regression coefficients, standard error and relative importance of each predictor from SAR models of range-size mean and variability (SD)

	SAR_avg_	SE	W_AIC_
Mean			
TSEA	0.423	0.098	0.999
PSEA	−0.069	0.036	0.690
ClimVel	0.245	0.048	1.000
Land	0.435	0.089	1.000
ClimRare	−0.048	0.042	0.415
ElevRange	−0.051	0.031	0.582
SD			
TSEA	−0.379	0.070	0.999
PSEA	0.000	0.025	0.269
ClimVel	−0.071	0.037	0.698
Land	−0.268	0.054	1.000
ClimRare	0.046	0.031	0.528
ElevRange	0.165	0.026	1.000

SAR_avg_, averaged standardised regression coefficient; SE, standard error; W_AIC_, summed Akaike weights; Parameters: TSEA, temperature seasonality; PSEA, precipitation seasonality; ClimVel, climate-change velocity; Land, land area; ClimRare, climate rarity; ElevRange, elevation range.

The randomisation analysis revealed strong geographical patterns in the distribution of areas with range-size assemblage characteristics deviating from random expectation (Fig. 1b). The large amount of spatial variation in number of records and variation in species richness (ranging from 1 to 6025 per cell in our data set) could potentially bias the range-size distribution patterns. However, the spatial patterns in the randomised maps did not indicate any strong effect of species richness or sampling biases on observed range-size frequency distributions. Conversely, overlaying a map of glacial extent at 21 kyr (Peltier 1994) showed that the area where mean range sizes are higher than expected by chance in North America coincides with regions formerly covered by glaciers. SD of range size was also lower than expected in glaciated areas, except in a smaller glaciated region in southern South America (Fig. 1b).

Four main spectrum types resulted from classifying grid cells according to the mean, SD, skewness and kurtosis of the frequency distribution of range sizes observed in each cell (Fig. 3a, b). The spectrum types were generally clustered into geographical regions, and differed significantly in their environmental characteristics (Fig. 3c). Notably, one range-size spectrum type (Type 1) with large assemblage range-size means and small variability was primarily associated with large land area, resulting in representation mainly in the Amazon and northern regions of North America, although in the latter region it was also associated with previously glaciated areas, high temperature seasonality and Late Quaternary climate-change velocity (Fig. 3). Type 2 was similar to Type 1 in mean and variability characteristics, but had high kurtosis and negative skewness. It was mostly confined to an area in eastern North America and characterised by significantly lower temperature and precipitation seasonality and higher climate rarity than Type 1 (Fig. 3). Two common range-size spectrum types (Types 3 and 4) had relatively small mean range sizes. They were both also characterised by high variability, and had low land area, low Late Quaternary climate-change velocity, large elevation range and high climatic rarity, with one (Type 4) being more extreme in all these variables than the other (Fig. 3).

The expected future climate-change velocities (in mean annual temperature for 2080s under the A1B scenario) differed among the range-size spectrum types (Fig. 3c). The greatest velocities were expected for the types also characterised by the largest range sizes, greatest Late Quaternary climate-change velocity, and highest temperature seasonality. On the other hand, regions with past and present stable climates, where small-ranged species were concentrated, were expected to experience lower future climate-change velocities (Fig. 3c).

## Discussion

Across the New World, the distribution of plant geographical range size significantly shifted in both mean and shape. These changes were driven by geographical variation in both short- and long-term climate stability and habitat area, with the former having a somewhat stronger overall effect (Tables 1, 2). The northward increase in mean range size found in North America is concordant with Rapoport's rule (Stevens 1989). In contrast, the pattern in South America was reversed, with mean range size decreasing southward away from the equator, as also observed in other less-diverse organism groups (e.g. Hawkins & Diniz-Filho 2006). Temperature seasonality, Late Quaternary climate-change velocity and land area were all positively related to mean range size and emerged as the most important variables in explaining the overall pattern (Table 2, Fig. 2). The effect of hard boundaries on ranges has been used for creating null-models of range-size distribution and driven a lot of the debate on species richness patterns and Rapoport's rule (the mid-domain effect, Colwell & Hurtt 1994). Our land area measure captures a similar effect on the distribution of range size, namely area constraints on the potential of species for range expansion, although it is here considered as an explanatory factor rather than integrated into a null model. Our findings indicate that it is indeed an important factor, although in combination with the effects of climate stability in both the short- and long term.

The low importance of climate rarity relative to land area in explaining mean range size was consistent with many small-ranged species being mainly dispersal limited rather than climatically limited (Baselga *et al*. 2012). Variability (SD) in range size was generally inversely related to mean range size, with little variability where means were high and vice versa (Fig. 1a). As for mean range size, land area and temperature seasonality were also important predictors of range-size variability, with less variability where large land areas were available, and where temperature varied much among seasons. Additionally, there was high variability where elevation range was high, i.e. in mountainous areas (Table 2, Fig. 2). Variability in range size declined with increasing Late Quaternary climate-change velocity, but the effect was relatively weak (Table 2, Fig. 2). Observed lower variability and higher mean in range sizes in areas that are climatically unstable was consistent with a differential selection for broad-ranged generalist species, supported by recent findings on niche breadth of North American trees (Morin & Lechowicz 2013; see also Slatyer *et al*. 2013).

The role played by short- and long-term climatic stability was emphasised when mapping areas with higher or lower assemblage range-size mean or variability than expected from the range-size frequency distribution of all recorded plant species in the New World (Fig. 1b). Areas where range-size assemblages had higher mean and lower variability than the random expectation coincided with areas of highest temperature seasonality and highest Late Quaternary climate-change velocity (Fig. 2). Strikingly, the pattern also largely matched the limits of the massive Last Glacial ice sheets in North America (Fig 1b). This finding shows that part of the link to climatic stability may reflect the physical effect of glaciers excluding all macroscopic living organisms (Brown 1995; Davies *et al*. 2009) rather than climate *per se*. Previous arguments de-emphasising the role of glaciers have required a step-like gradient in observed range-size patterns to support a glaciation effect (Gaston *et al*. 1998), and our results provide evidence that such an effect is indeed present in the New World flora. Nevertheless, climate-change velocity was still important for range-size mean and variability when excluding previously glaciated areas in the SAR models (Table S5), consistent with Jansson (2003) and Sandel *et al*. (2011).

While we found that climate stability and habitat area were both important for the overall patterns of the mean and variability in range size, our results showed that the relative importance of the two broad mechanisms varies across regions, depending on their specific environmental conditions. Arguably, these shifts in relative importance may contribute to the lack of consensus in the ongoing debate of which processes determine range-size distribution patterns. For instance, the inconsistent relationship between range size and temperature seasonality away from the equator towards the north and the south, respectively, has been argued to be evidence contrary to Rapoport's rule and the importance of climate stability in driving range-size patterns (Rohde 1996; Gaston *et al*. 1998; Weiser *et al*. 2007). Classifying cells into range-size spectrum types shed light on the joint effects of different interacting drivers on the assemblage of range sizes, and notably helped explain the asymmetrical latitudinal patterns in the two hemispheres. Spectrum types 1 and 2, with the largest mean range sizes and lowest SD, are mostly associated to previously glaciated areas of northern North America, pointing to a joint effect of temporally unstable climates and large habitat areas. Such conditions may promote large range sizes via climate-driven extinction of small-ranged species, seasonality-driven selection for climatic generalism and opportunity for the remaining species to spread widely into large areas of suitable habitat (Fig. 3). Land area *per se* can also result in spectrum Type 1, as seen by its prevalence in the Amazon. Spectrum Type 2 is especially associated with the mountainous Appalachian region of eastern North America. It shares high means and low SDs with spectrum Type 1, consistent with their similar land area and climate-change velocity characteristics. The high kurtosis of Type 2 is caused by a high incidence of broad-ranged species with similar range sizes, possibly reflecting the predominance of temperate forest species that have expanded across much of eastern North America, at least partly from broadly distributed cryptic glacial refugia (Soltis *et al*. 2006). However, the exceptionally negative skewness (Fig. 3, Fig. S4) also reflects the presence of small-range species (e.g. *Abies fraseri* (Pursh) Poir., *Aesculus* spp., *Magnolia* spp., *Oxydendrum arboretum* (L.) DC and *Tsuga caroliniana* Engelm.), perhaps reflecting dispersal-limited postglacial expansions and mountain-habitat species.

The last two spectrum types, 3 and 4, are characterised by generally higher climate stability and smaller habitat areas, with consequently lower mean and higher SD in observed range-size assemblages. The most extreme of the two, Type 4, with the smallest mean range sizes, is found principally in the Andes, Central America and Brazilian Atlantic Rainforest region, suggesting that the appropriate conditions for small-ranged species to arise and accumulate are created by the interaction of particularly stable long-term climate with relatively small land areas, high climatic rarity, and large elevation ranges (Table 2, Fig. 3). As no known mechanism prevents broad-ranged species from coexisting with endemics in such areas, this would explain the extremely high range-size variability and lesser negative skew of types 3 and 4 relative to the remaining spectrum types (Fig. 3, Fig. S2, S4).

Taken together, the shifting relative importance of drivers of range-size patterns can account for the reversal of Rapoport's rule south of the equator. Range-size spectrum types where habitat area plays a more important role are mostly represented in tropical regions, resulting in a decrease of mean range size towards the south in spite of the increasing temperature seasonality. This is not surprising, given the much less pronounced gradient both in temperature seasonality and climate-change velocity in this region compared to the Nearctic. Additional models of range-size mean and SD for the two regions support these findings, with climate stability overall being more important in the Nearctic than in the Neotropical region, where climate stability and habitat area are of equal importance (Tables S6, S7).

Data sets of the size used here are not devoid of errors. We addressed the major data issues at different levels: revising and correcting taxonomic names, validating coordinates, and excluding records from plantations (see Enquist 2009). Also, in spite of the high proportion of rare species in the data set, they did not bias the results, since excluding them did not change the conclusions. Data checking was mostly automated and not perfect, but given the magnitude of the data set, we are confident the results presented accurately reflect the true patterns. To our knowledge, the next largest data set is from Missouri Botanical Garden, with ∼3.9 mio. records out of the ∼ 10.9 mio. in BIEN before data checking.

Although the mechanisms tested here represent a broad range of hypotheses, other factors might influence patterns of range-size distribution. Besides latitude and productivity (but see Methods), evolutionary history is another proposed driver of range size. Older lineages have been proposed to have larger ranges due to the larger amount of time to spread (the age and area hypothesis, Willis 1922). However, Willis’ hypothesis has been widely rejected due to numerous examples of young lineages with broad ranges and old lineages with narrow distributions (cf. Gaston 1998). Although a phylogenetic analysis is outside the scope of the present study, the data set provides the means for exploring such evolutionary hypotheses in the future.

An important finding for biodiversity conservation is that the greatest future climate-change velocities are expected in regions which have experienced the most unstable climates in the past and have the highest temperature seasonality. In other words, the greatest spatiotemporal climate shifts are expected in areas where the biota is expected to be most resilient to climate change due to past and present sorting processes, causing constituent species to be climate generalists and/or good dispersers. On the other hand, regions with past and present stable climates, where small-ranged species are concentrated, are expected to experience lower future climate-change velocities (Fig. 3). However, the magnitude of future changes might still exceed what species in these regions may tolerate (Dullinger *et al*. 2012). Finally, the high importance of habitat area raises concerns over increasing habitat losses due to human land use.

In summary, creation of the BIEN data set has allowed us, for the first time, to map and analyse macroecological patterns of range-size distributions at spatial scales encompassing the entire New World flora. Importantly, we find that geographical variation in species range sizes is jointly determined by long- and short-term climatic stability, in addition to land area. These results suggest that species’ range sizes have been shaped by a combination of processes. Observed patterns of range-size distribution are consistent with the importance of climatic evolution of niche breadth, diversification rates, and effects of areal constraints on range expansions. Importantly, the relative importance of these drivers changes across space. Lastly, glaciation history appears to have left a strong imprint on the geographical distribution of assemblage range characteristics. Although the coincidence of regions of predominantly large ranges and high future climate-change velocity points to some resilience to environmental change, the strong links between range-size patterns and climate stability and area indicate that ongoing climate change and habitat loss due to land-use change will likely have profound influences on species distributions and diversity in the future.
