# Supplementary material for: Habitat area and climate stability determine geographical variation in plant species range sizes
Source: Ecol Lett. 2013 Oct 3;16(12):1446–54. doi: 10.1111/ele.12184 (PMC4068282; doi:10.1111/ele.12184)
Supplement: Supplementary file 1 [file ele0016-1446-SD1.docx]

**SUPPORTING INFORMATION**

Additional Supporting Information may be downloaded via the online version of this article at Wiley Online Library (www.ecologyletters.com).

As a service to our authors and readers, this journal provides supporting information supplied by the authors. Such materials are peer-reviewed and may be re-organized for online delivery, but are not copy-edited or typeset. Technical support issues arising from supporting information (other than missing files) should be addressed to the authors.
