# Supplementary material for: Habitat area and climate stability determine geographical variation in plant species range sizes
Source: Ecol Lett. 2013 Oct 3;16(12):1446–54. doi: 10.1111/ele.12184 (PMC4068282; doi:10.1111/ele.12184)
Supplement: Supplementary file 2 [file ele0016-1446-SD2.pdf]

**Supporting Information**

**Figure S1** Bivariate relationships between potential predictors and range-size mean and standard deviation. Predictors: precipitation seasonality (PSEA) and climate rarity (ClimRare, unitless). Linear and Gaussian local (LOESS, fitted with span = 0.75 and a quadratic term) regressions were fitted for all cells with at least one recorded species.

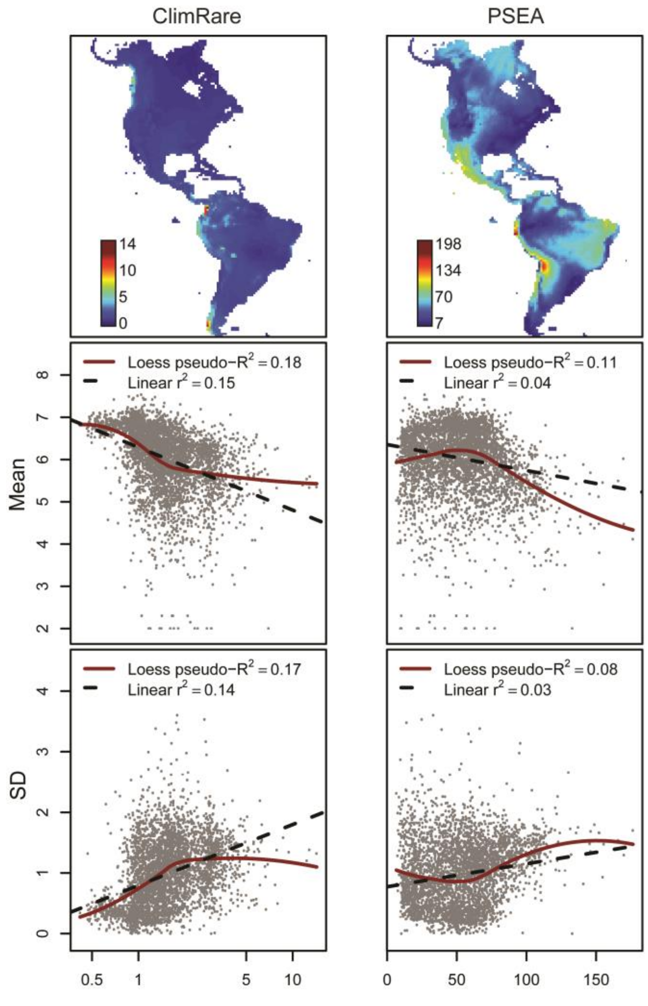

**Figure S2** Maps for (a) range-size median ($km^2$), skewness and kurtosis of New World plants; and (b) deviations of range-size spectra from random expectation. Cells with a range-size spectra value greater or lower than expected given the observed species richness are colored red or blue, respectively. Black line delimits glaciated areas during the Last Glacial Maximum.

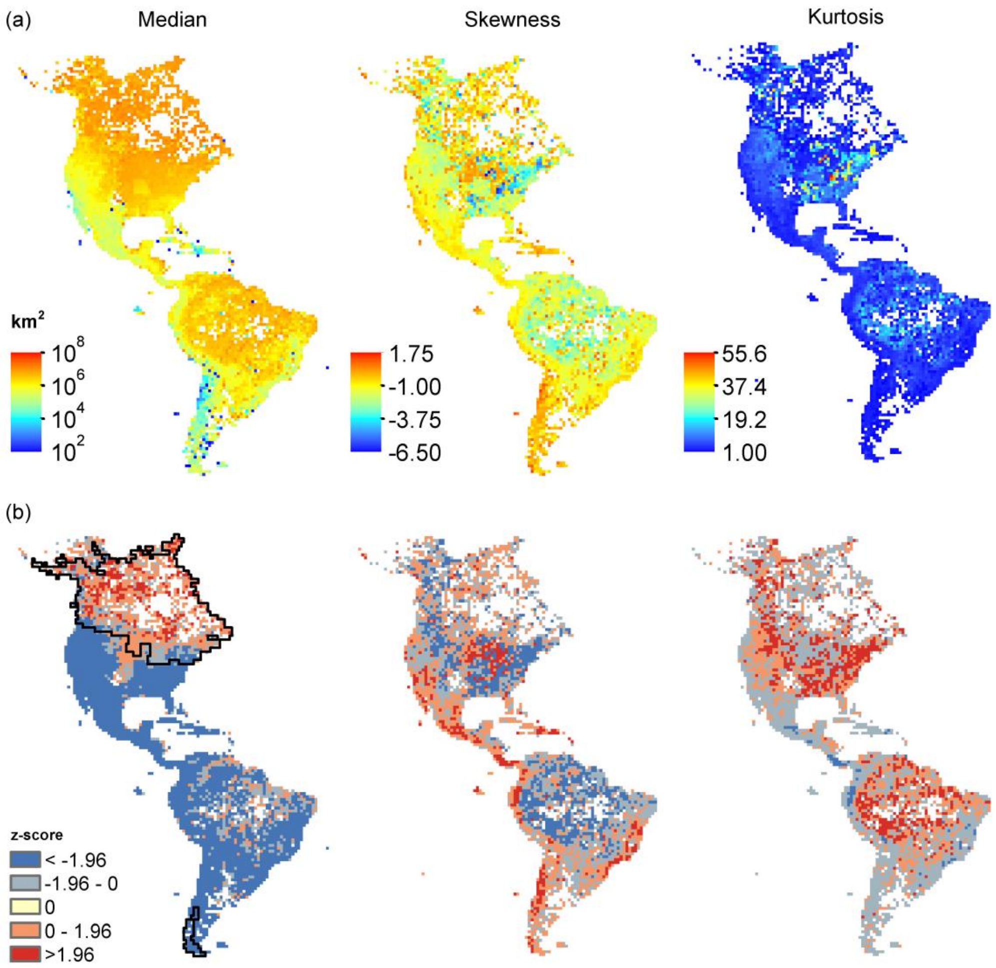

**Figure S3** Result of the k-means cluster analysis, showing the four range-size spectra types mapped in Fig. 3 in the main text.

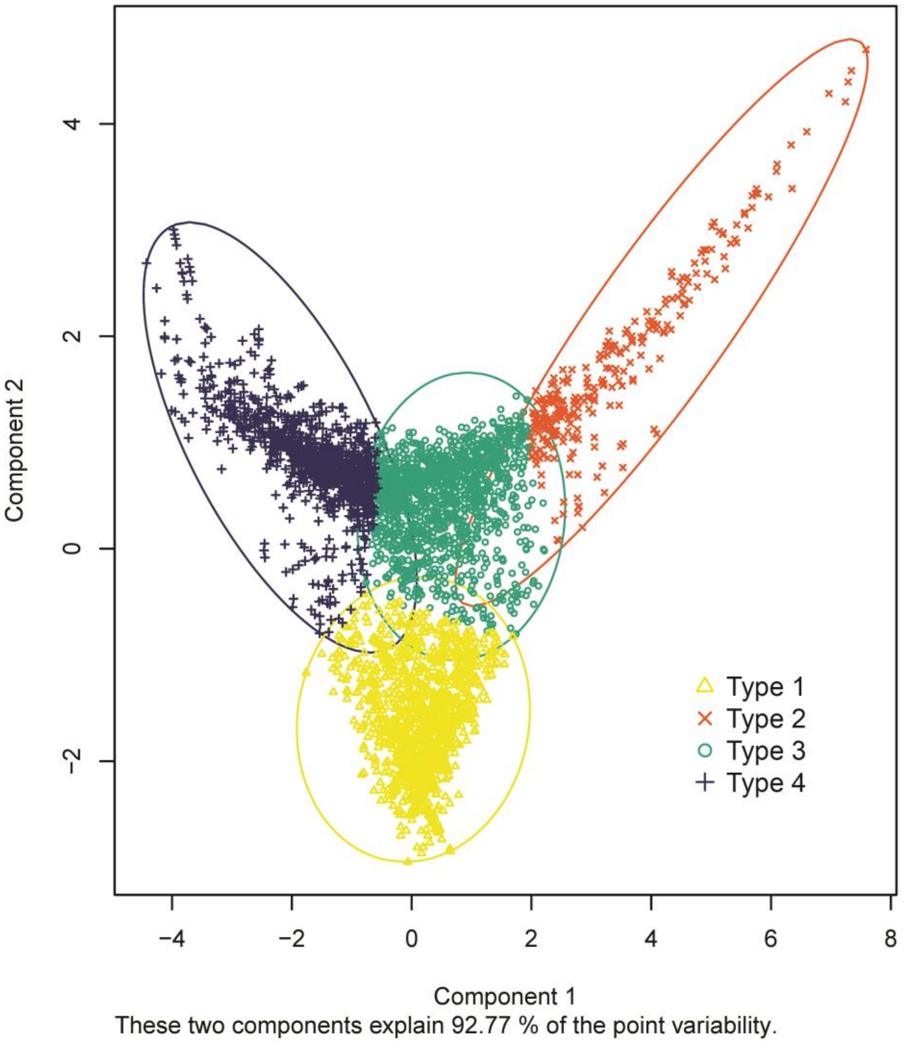

**Figure S4** Overlaid range-size frequency distributions for a random sample of 250 cells within each of the four spectrum types mapped in Fig. 3 and 250 random cells across the whole study region. Range sizes are in $\log_{10}$ scale. The figure illustrates the typical histogram of range sizes within each spectrum type. For instance, the high mean, low variability and less negative kurtosis of Type 1 is arguably caused by the dominance of broad-ranged species and lack of small-ranged species.

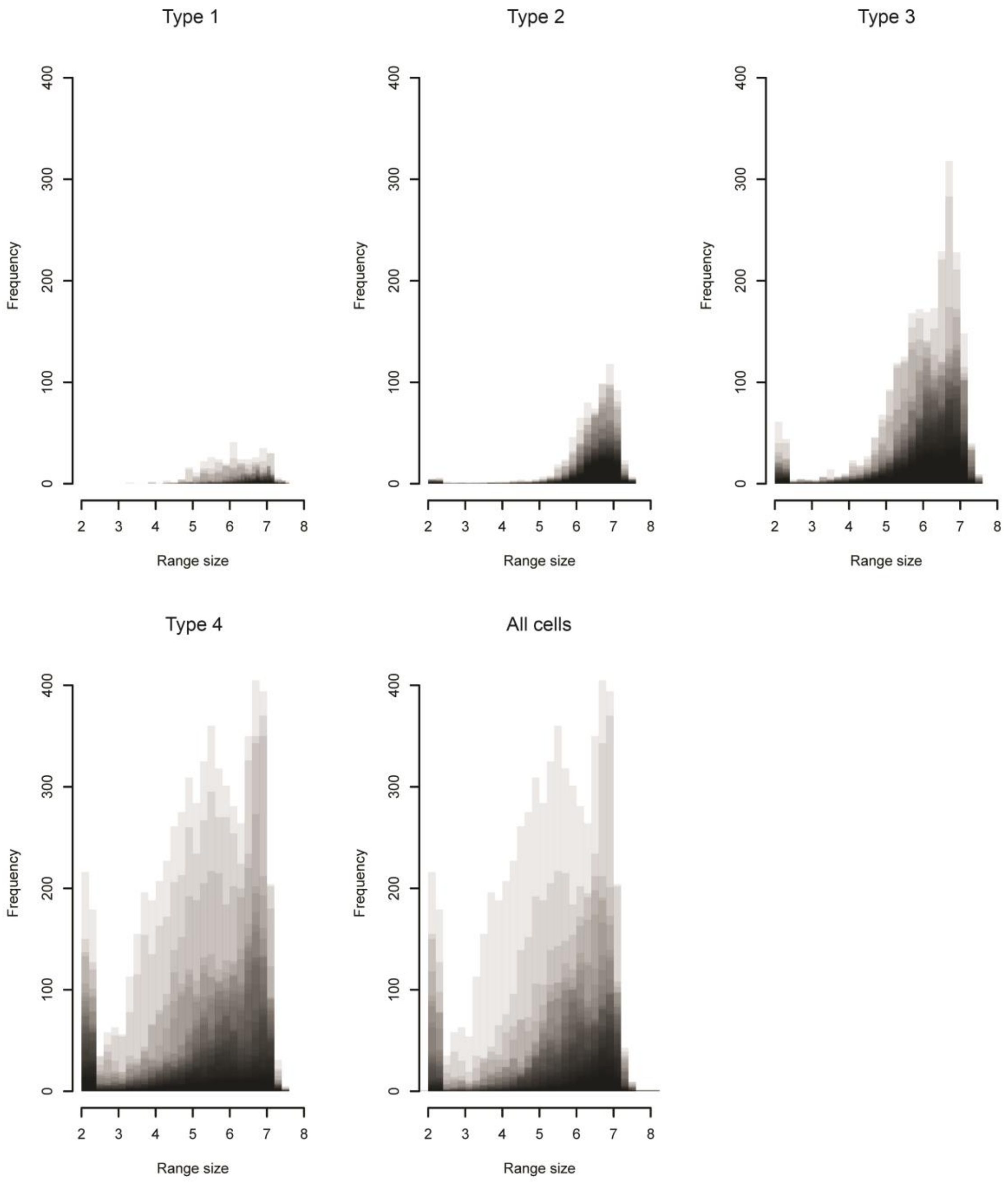

**Table S1** Summary results for full OLS models explaining the mean and standard deviation (SD) patterns for $log_{10}$-transformed range sizes. AIC: Akaike's information criterion, minRSA: residual spatial autocorrelation (summed absolute Moran's I values of the first 20 distance classes), Max I: maximum Moran's I in the first 20 distance classes, $R^2$: variation explained in full models, Vc: unique contribution of climate stability, $V_H$: unique contribution of habitat area, $V_{CH}$: shared effect of climate stability and habitat area.

| | AIC | minRSA | Max I | $R^2$ | $V_C$ | $V_H$ | $V_{CH}$ |
|---|---|---|---|---|---|---|---|
| **Mean** | 7,445 | 4.458 | 0.511 | 0.387 | 0.101 | 0.079 | 0.207 |
| **SD** | 4,157 | 1.710 | 0.248 | 0.404 | 0.159 | 0.045 | 0.200 |

**Table S2** Averaged standardized regression coefficients ($OLS_{avg}$), standard error (SE) and relative importance (summed Akaike weights, $W_{AIC}$) from OLS models of range-size mean and variability (standard deviation). Parameters: temperature seasonality (TSEA), precipitation seasonality (PSEA), climate-change velocity (ClimVel), land area (Land), climate rarity (ClimRare), and elevation range (ElevRange).

| | $OLS_{avg}$ | SE | $W_{AIC}$ |
|---|---|---|---|
| **Mean** | | | |
| TSEA | 0.602 | 0.029 | 1.000 |
| PSEA | -0.127 | 0.021 | 1.000 |
| ClimVel | -0.092 | 0.032 | 0.952 |
| Land | 0.421 | 0.023 | 1.000 |
| ClimRare | -0.123 | 0.026 | 1.000 |
| ElevRange | -0.287 | 0.027 | 1.000 |
| **SD** | | | |
| TSEA | -0.554 | 0.018 | 1.000 |
| PSEA | 0.018 | 0.014 | 0.456 |
| ClimVel | 0.001 | 0.023 | 0.271 |
| Land | -0.232 | 0.015 | 1.000 |
| ClimRare | -0.018 | 0.018 | 0.384 |
| ElevRange | 0.156 | 0.016 | 1.000 |

**Table S3** Results from SAR and OLS models of range-size mean and variability (standard deviation) with varying radius used for land area. Abbreviations as in Table S2.

| | **Land radius = 800 km** | | | | | | **Land radius = 1300 km** | | | | | |
|---|---|---|---|---|---|---|---|---|---|---|---|---|
| | **SAR** | | | **OLS** | | | **SAR** | | | **OLS** | | |
| | **$SAR_{avg}$** | **SE** | **$W_{AIC}$** | **$OLS_{avg}$** | **SE** | **$W_{AIC}$** | **$SAR_{avg}$** | **SE** | **$W_{AIC}$** | **$OLS_{avg}$** | **SE** | **$W_{AIC}$** |
| **Mean** | | | | | | | | | | | | |
| TSEA | 0.405 | 0.100 | 0.997 | 0.593 | 0.028 | 1.000 | 0.412 | 0.100 | 0.998 | 0.593 | 0.029 | 1.000 |
| PSEA | -0.066 | 0.036 | 0.654 | -0.106 | 0.021 | 1.000 | -0.068 | 0.036 | 0.681 | -0.114 | 0.021 | 1.000 |
| ClimVel | 0.242 | 0.050 | 1.000 | -0.035 | 0.032 | 0.400 | 0.247 | 0.048 | 1.000 | -0.057 | 0.032 | 0.635 |
| Land | 0.327 | 0.065 | 1.000 | 0.346 | 0.021 | 1.000 | 0.414 | 0.083 | 1.000 | 0.382 | 0.022 | 1.000 |
| ClimRare | -0.043 | 0.042 | 0.382 | -0.118 | 0.026 | 1.000 | -0.050 | 0.042 | 0.426 | -0.122 | 0.026 | 1.000 |
| ElevRange | -0.064 | 0.031 | 0.741 | -0.311 | 0.025 | 1.000 | -0.053 | 0.031 | 0.607 | -0.282 | 0.028 | 1.000 |
| **SD** | | | | | | | | | | | | |
| TSEA | -0.331 | 0.073 | 0.984 | -0.559 | 0.019 | 1.000 | -0.367 | 0.072 | 0.998 | -0.558 | 0.018 | 1.000 |
| PSEA | -0.004 | 0.025 | 0.271 | 0.011 | 0.015 | 0.333 | 0.000 | 0.025 | 0.269 | 0.015 | 0.015 | 0.381 |
| ClimVel | -0.068 | 0.037 | 0.661 | -0.034 | 0.023 | 0.538 | -0.075 | 0.037 | 0.739 | -0.024 | 0.023 | 0.393 |
| Land | -0.219 | 0.038 | 1.000 | -0.184 | 0.015 | 1.000 | -0.229 | 0.050 | 1.000 | -0.202 | 0.015 | 1.000 |
| ClimRare | 0.043 | 0.031 | 0.486 | -0.018 | 0.018 | 0.376 | 0.049 | 0.031 | 0.554 | -0.016 | 0.018 | 0.358 |
| ElevRange | 0.180 | 0.026 | 1.000 | 0.182 | 0.019 | 1.000 | 0.166 | 0.026 | 1.000 | 0.165 | 0.017 | 1.000 |

**Table S4** Results from SAR and OLS models of range-size mean and variability (standard deviation) excluding rare species (defined as species with range size = 10 000 $km^2$). Abbreviations as in Table S2.

| | **SAR** | | | **OLS** | | |
|---|---|---|---|---|---|---|
| | **$SAR_{avg}$** | **SE** | **$W_{AIC}$** | **$OLS_{avg}$** | **SE** | **$W_{AIC}$** |
| **Mean** | | | | | | |
| TSEA | 0.371 | 0.078 | 1.000 | 0.493 | 0.024 | 1.000 |
| PSEA | -0.059 | 0.029 | 0.733 | -0.117 | 0.017 | 1.000 |
| ClimVel | 0.180 | 0.041 | 1.000 | -0.065 | 0.026 | 0.879 |
| Land | 0.305 | 0.071 | 1.000 | 0.309 | 0.019 | 1.000 |
| ClimRare | -0.026 | 0.034 | 0.332 | -0.114 | 0.021 | 1.000 |
| ElevRange | -0.058 | 0.025 | 0.837 | -0.259 | 0.023 | 1.000 |
| **SD** | | | | | | |
| TSEA | -0.297 | 0.053 | 0.999 | -0.415 | 0.016 | 1.000 |
| PSEA | 0.013 | 0.021 | 0.308 | 0.034 | 0.012 | 0.951 |
| ClimVel | -0.074 | 0.030 | 0.876 | -0.037 | 0.019 | 0.708 |
| Land | -0.237 | 0.043 | 1.000 | -0.197 | 0.014 | 1.000 |
| ClimRare | 0.029 | 0.026 | 0.416 | 0.011 | 0.016 | 0.327 |
| ElevRange | 0.115 | 0.022 | 1.000 | 0.118 | 0.017 | 1.000 |

**Table S5** Results from SAR and OLS models of range-size mean and variability (standard deviation) excluding regions that were glaciated during the Last Glacial Maximum. Abbreviations as in Table S2.

| | SAR | | | OLS | | |
|---|---|---|---|---|---|---|
| | $SAR_{avg}$ | SE | $W_{AIC}$ | $OLS_{avg}$ | SE | $W_{AIC}$ |
| **Mean** | | | | | | |
| TSEA | 0.250 | 0.135 | 0.670 | 0.379 | 0.041 | 1.000 |
| PSEA | -0.058 | 0.040 | 0.513 | -0.033 | 0.025 | 0.465 |
| ClimVel | 0.348 | 0.060 | 1.000 | -0.043 | 0.046 | 0.364 |
| Land | 0.409 | 0.094 | 0.999 | 0.488 | 0.027 | 1.000 |
| ClimRare | -0.060 | 0.050 | 0.438 | -0.061 | 0.031 | 0.735 |
| ElevRange | -0.062 | 0.037 | 0.603 | -0.352 | 0.029 | 1.000 |
| **SD** | | | | | | |
| TSEA | -0.410 | 0.087 | 1.000 | -0.491 | 0.026 | 1.000 |
| PSEA | -0.026 | 0.026 | 0.374 | -0.011 | 0.017 | 0.313 |
| ClimVel | -0.099 | 0.042 | 0.850 | -0.023 | 0.031 | 0.330 |
| Land | -0.252 | 0.055 | 1.000 | -0.251 | 0.018 | 1.000 |
| ClimRare | 0.057 | 0.034 | 0.594 | -0.027 | 0.020 | 0.468 |
| ElevRange | 0.174 | 0.029 | 1.000 | 0.187 | 0.019 | 1.000 |

**Table S6** Averaged standardized regression coefficients, standard error (SE) and relative importance from SAR models of range-size mean and standard deviation in the Nearctic and Neotropic regions. The division was set at 20° of northern latitude, the border between the Nearctic and Neotropical biogeographic realms, coinciding with the latitude where the minimum mean range sizes are found for plants and other taxa (Blackburn & Gaston 1996)[1]. Abbreviations as in Table S2. The results from these models (see also Table S6) support and emphasize the findings of the classification analysis: In the Nearctic, range sizes appear to be more influenced by climate stability than habitat area compared to the Neotropics, where habitat area plays a relatively stronger role. Note the reversal of Rapoport's rule in the Neotropics, where mean range size is negatively related to TSEA, arguably due to the effect of smaller land area restricting the species' ranges towards the south.

| | Nearctic | | | Neotropic | | |
|---|---|---|---|---|---|---|
| | $SAR_{avg}$ | SE | $W_{AIC}$ | $SAR_{avg}$ | SE | $W_{AIC}$ |
| **Mean** | | | | | | |
| TSEA | 0.930 | 0.101 | 1.000 | -1.484 | 0.280 | 0.999 |
| PSEA | -0.178 | 0.051 | 0.988 | -0.008 | 0.045 | 0.272 |
| ClimVel | 0.103 | 0.052 | 0.761 | 0.517 | 0.085 | 1.000 |
| Land | 0.069 | 0.081 | 0.355 | 0.498 | 0.090 | 1.000 |
| ClimRare | 0.085 | 0.054 | 0.541 | -0.075 | 0.056 | 0.477 |
| ElevRange | 0.076 | 0.042 | 0.673 | -0.096 | 0.045 | 0.766 |
| **SD** | | | | | | |
| TSEA | -0.458 | 0.080 | 0.999 | 0.512 | 0.173 | 0.907 |
| PSEA | 0.127 | 0.040 | 0.981 | -0.083 | 0.029 | 0.949 |
| ClimVel | -0.080 | 0.040 | 0.737 | -0.140 | 0.058 | 0.875 |
| Land | -0.135 | 0.065 | 0.757 | -0.210 | 0.056 | 0.998 |
| ClimRare | 0.030 | 0.047 | 0.312 | 0.012 | 0.038 | 0.279 |
| ElevRange | 0.022 | 0.037 | 0.339 | 0.229 | 0.037 | 1.000 |

[1]Blackburn, T.M. & Gaston, K.J. (1996). Spatial patterns in the geographic range sizes of bird species in the New World. *Philos. Trans. R. Soc. Lond., Ser. B: Biol. Sci.*, 351, 897-912.

**Table S7** Variation partitioning of the two broad mechanisms, climate variability and habitat area, in the Nearctic and Neotropic regions, and areas that were non-glaciated during the Last Glacial Maximum. $V_{total}$: variation ($R^2$) explained in full models, unique contribution of climate stability ($V_C$) and habitat area ($V_H$) and their combined effect ($V_{CH}$).

| | SAR | | | | OLS | | | |
|---|---|---|---|---|---|---|---|---|
| | $V_{total}$ | $V_C$ | $V_H$ | $V_{CH}$ | $V_{total}$ | $V_C$ | $V_H$ | $V_{CH}$ |
| **Nearctic** | | | | | | | | |
| Mean | 0.545 | 0.428 | 0.004 | 0.114 | 0.557 | 0.363 | 0.012 | 0.183 |
| SD | 0.360 | 0.185 | -0.001 | 0.176 | 0.373 | 0.165 | 0.008 | 0.200 |
| **Neotropic** | | | | | | | | |
| Mean | 0.414 | 0.153 | 0.002 | 0.258 | 0.448 | 0.158 | 0.033 | 0.256 |
| SD | 0.213 | 0.046 | 0.039 | 0.127 | 0.227 | 0.051 | 0.052 | 0.125 |
| **Non-glaciated** | | | | | | | | |
| Mean | 0.227 | 0.015 | 0.092 | 0.120 | 0.290 | 0.034 | 0.113 | 0.143 |
| SD | 0.262 | 0.109 | 0.053 | 0.100 | 0.270 | 0.111 | 0.060 | 0.099 |

**Table S8** Contributors of data from plots in the BIEN2 database used in the study.

| Source | Observations | Dataset owner |
|---|---|---|
| Forest Inventory and Analysis National Program (FIA) | 1827366 | |
| VegBank | 419112 | |
| Carolina Vegetation Survey | 239260 | |
| TEAM Permanent Plots | 11169 | |
| Madidi Transects | 10911 | Peter Jørgensen |
| Center for Tropical Forest Science (CTFS) | 7015 | |
| Madidi Permanent Plots | 2129 | Peter Jørgensen |
| SALVIAS | | |
| Gentry Transect Dataset | 9488 | Al Gentry |
| RAINFOR - 0.1 ha Madre de Dios, Peru | 8166 | Percy Nuñez |
| RAINFOR - 1 ha Peru | 3939 | |
| Noel Kempff Forest Plots | 1694 | Tim Killeen |
| Boyle Transects | 1658 | Brad Boyle |
| Noel Kempff Savanna Plots | 630 | Tim Killeen |
| OTS Transects | 623 | Brad Boyle |
| DeWalt Bolivia forest plots | 283 | Saara DeWalt |
| La Selva Secondary Forest Plots | 180 | Susan G. Letcher |
| Pilon Lajas Treeplots Bolivia | 164 | |
| ACA Amazon Forest Inventories | 99 | |
| Bonifacino Forest Transects | 86 | Mauricio Bonifacio |

**Table S9** Contributors of specimen data in the BIEN2 database used in the study. Data provided through GBIF, REMIB-CONABIO, SpeciesLink and individual herbaria. Full herbarium names, institutions and locations available at http://sciweb.nybg.org/science2/IndexHerbariorum.asp.

| Herbarium | Observations | Herbarium | Observations | Herbarium | Observations |
|---|---|---|---|---|---|
| MO | 2666166 | NY | 502580 | US | 275861 |
| INB | 167135 | INPA | 145142 | CR | 134623 |
| ASU | 119680 | OSC | 107013 | IB | 105991 |
| XAL | 100254 | TEX | 86936 | IEB | 79025 |
| COL | 73102 | MNHN | 66587 | AAU | 50032 |
| UNM | 41202 | CHSC | 39489 | ENCB | 37577 |
| CICY | 36348 | LL | 35398 | FURB | 33438 |
| MEXU | 32758 | CS | 31093 | IPA | 29979 |
| IAC | 29810 | FTG | 29738 | MA | 27712 |
| GH | 25101 | UNCC | 24656 | K | 14348 |
| S | 12657 | NMC | 12510 | ACAD | 12343 |
| MBML | 10708 | HAM | 10204 | USP | 9577 |
| HCIB | 9523 | NEBC | 9397 | BCMEX | 8239 |
| AAS | 7580 | UFS | 7139 | UVSC | 6397 |
| UAMIZ | 6367 | NMCR | 6063 | UFRN | 5969 |
| AMES | 5590 | ZSS | 5061 | A | 4253 |
| USON | 4247 | LI | 4173 | NCU | 4100 |
| LEB | 3781 | HOH | 3541 | O | 3189 |
| UGDA | 2606 | IZTA | 2585 | GOET | 2176 |
| SANT | 1868 | NSW | 1757 | MICH | 1587 |
| SD | 1367 | Z | 1064 | OHN | 1054 |
| FH | 1022 | IBUG | 624 | GB | 606 |
| SALA | 558 | HUAZ | 541 | SEV | 541 |
| UNL | 521 | DAO | 507 | CAS | 499 |
| SLPM | 482 | ND | 384 | SNM | 355 |
| BC | 338 | HUAA | 337 | RSA | 321 |
| HYO | 296 | STU | 267 | FCO | 259 |
| BG | 250 | ISC | 247 | LD | 247 |
| F | 232 | NCSC | 214 | INIF | 210 |
| QFA | 191 | CHAPA | 184 | QUE | 172 |
| MGC | 172 | MT | 164 | COA | 144 |
| ANSM | 142 | DUKE | 134 | BH | 119 |
| TRT | 116 | CAN | 116 | SASK | 111 |
| UC | 110 | NLU | 109 | NWOSU | 100 |

| Herbarium | Observations |
|---|---|
| INEGI | 88 |
| GEO | 77 |
| HUMO | 63 |
| P | 56 |
| ABH | 37 |
| WIS | 36 |
| DCH | 33 |
| C | 30 |
| MMMN | 27 |
| NCAS | 24 |
| UFMA | 23 |
| GDA | 19 |
| CANB | 17 |
| ALTA | 15 |
| ZEA | 14 |
| FCME | 12 |
| BIO | 11 |
| TLXM | 10 |
| WCUH | 8 |
| MUB | 8 |
| PAC | 7 |
| TUC | 6 |
| EMMA | 5 |
| ODU | 4 |
| UMO | 4 |
| DES | 4 |
| VPI | 3 |
| EBUM | 3 |
| SMS | 3 |
| POM | 3 |
| M | 2 |
| UADY | 2 |
| TENN | 2 |
| NO | 2 |
| ANA | 1 |
| GDAC | 1 |
| OAC | 1 |
| GA | 1 |

| Herbarium | Observations |
|---|---|
| ASC | 84 |
| MOR | 69 |
| WIN | 60 |
| CODAGEM | 45 |
| V | 37 |
| ARAN | 35 |
| MTMG | 33 |
| VAL | 30 |
| PENN | 26 |
| IMSSM | 24 |
| KAG | 21 |
| ALBC | 19 |
| UNB | 17 |
| UAS | 15 |
| WAT | 13 |
| COCA | 11 |
| BR | 11 |
| BOON | 9 |
| G | 8 |
| B | 7 |
| PH | 7 |
| ASTC | 6 |
| NA | 5 |
| OKL | 4 |
| PACA | 4 |
| USF | 3 |
| VCU | 3 |
| UBC | 3 |
| ILLS | 3 |
| MU | 2 |
| NCC | 2 |
| TRH | 2 |
| FSU | 2 |
| OS | 2 |
| OXF | 1 |
| SWBR | 1 |
| AUA | 1 |
| DPU | 1 |

| Herbarium | Observations |
|---|---|
| JEPS | 82 |
| ECON | 65 |
| HSS | 57 |
| ARIZ | 45 |
| CIMI | 36 |
| JBAG | 34 |
| BM | 31 |
| TRTE | 29 |
| ZT | 25 |
| TI | 24 |
| CIQR | 19 |
| CIIDIR | 18 |
| UWO | 16 |
| NSPM | 14 |
| DS | 12 |
| CU | 11 |
| USCH | 10 |
| MISS | 8 |
| NMR | 8 |
| UB | 7 |
| KSC | 7 |
| BCN | 6 |
| FHO | 4 |
| RBR | 4 |
| CHAP | 4 |
| VT | 3 |
| BISH | 3 |
| SMU | 3 |
| VSC | 3 |
| CM | 2 |
| MUR | 2 |
| NDA | 2 |
| UFRJ | 2 |
| DAV | 2 |
| VMIL | 1 |
| KANU | 1 |
| MHA | 1 |
| KBSMS | 1 |

| Herbarium | Observations |
|---|---|
| DEK | 1 |
| COFC | 1 |
| IPB | 1 |
| LE | 1 |
| MH | 1 |

| Herbarium | Observations |
|---|---|
| HNT | 1 |
| CLEMS | 1 |
| KESC | 1 |
| CHL | 1 |
| FLAS | 1 |

| Herbarium | Observations |
|---|---|
| CORD | 1 |
| UPEI | 1 |
| CHRB | 1 |
| LOMA | 1 |
